# Robotic-Arm-Assisted Versus Manual Total Knee Arthroplasty: A Comparative Cohort Study of Gait and Postural Outcomes

**DOI:** 10.3390/jpm16070386

**Published:** 2026-07-19

**Authors:** Dimitris Koukoulias, Eustathios Kenanidis, Michael Potoupnis, Panagiotis V. Tsaklis, Eleftherios Tsiridis

**Affiliations:** 1 Department of Physiotherapy, General Hospital Papageorgiou, Ring Road Efkarpia, 56403 Thessaloniki, Greece; demkoukoulias@gmail.com; 2 Department of Physiotherapy, Faculty of Health Sciences, International Hellenic University, Alexander Campus, P.O. Box 141, 57400 Thessaloniki, Greece; 3 Academic Orthopaedic Department, Aristotle University Medical School, General Hospital Papageorgiou, Ring Road Efkarpia, 56403 Thessaloniki, Greece; ekenanidis@auth.gr (E.K.); mikepotoup@gmail.com (M.P.); 4 Tsiridis Orthopaedic Institute—ICAROS Clinic, 55535 Thessaloniki, Greece; 5 ErgoMech-Lab, Department of Physical Education and Sport Science, University of Thessaly, 42100 Trikala, Greece; ptsaklis@gmail.com; 6 Department of Molecular Medicine and Surgery, Karolinska Institute, SE-171 76 Solna, Sweden

**Keywords:** ROSA, robotic-assisted total knee arthroplasty, manual total knee arthroplasty, gait analysis, postural balance, functional alignment, patient-reported outcomes

## Abstract

**Background/objectives:** Evidence on gait and postural recovery after robotically assisted total knee arthroplasty (raTKA), particularly with the ROSA system, remains limited. This study compared early gait, postural, functional, and patient-reported outcomes (PROMs) between ROSA raTKA and manual TKA (mTKA), with PROMs also assessed at final follow-up. **Methods:** This comparative cohort study included primary TKA patients treated by a single senior surgeon using the same implant and alignment strategy. Patients underwent either ROSA raTKA or mTKA. At three months, a senior physiotherapist assessed quadricep and tibialis anterior maximum voluntary isometric strength (MVIS), centre-of-mass (CoM) kinematics, lower-limb weight distribution, timed-up-and-go (TUG), range of motion (ROM), KOOS Pain, activities of daily living (ADL), and quality of life (QoL). The same KOOS domains were compared at final follow-up. **Results:** Seventy primary TKAs were included: 46 raTKAs and 24 mTKAs. No intraoperative complications occurred. Groups were comparable for age, BMI, sex, grip strength, and preoperative KOOS. At three months, no significant differences were found in quadricep MVIS (*p* = 0.257), tibialis anterior MVIS (*p* = 0.327), CoM kinematics (*p* = 0.066), weight distribution (*p* = 0.189), TUG (*p* = 0.599), ROM (*p* = 0.165), or KOOS domains. At final follow-up, KOOS-ADL (*p* = 0.041) and QoL (*p* = 0.032) were better in the raTKA group, but after Holm–Bonferroni correction, they were no longer significant (QoL, *p* = 0.384; ADL, *p* = 0.451). **Conclusions:** ROSA raTKA showed comparable early gait and postural recovery to mTKA. The marginal differences in KOOS domains are exploratory, as they were no longer significant after multiple-comparisons correction.

## 1. Introduction

Conventional manual total knee arthroplasty (mTKA) has traditionally relied on standardized alignment principles, despite substantial inter-individual variability in knee anatomy, ligamentous laxity, and functional biomechanics. Robotically assisted total knee arthroplasty (raTKA) has been introduced to improve implant positioning accuracy and support intraoperative soft-tissue balancing [1]. Beyond the precision of component positioning, raTKA may enhance intraoperative ligament balancing by permitting controlled adjustments of bone resections and component placement based on soft-tissue gaps [2]. Rossi and Benazzo have detailed individualized alignment and ligament-balancing strategies utilizing the ROSA^®^ Knee System, whereas Tsai et al. reported a functional alignment, gap-balancing technique employing the MAKO robotic-arm system [3,4]. These investigations imply that robotic platforms could enable more precise intraoperative balancing; however, the extent to which these technical benefits translate into observable postoperative functional improvements remains uncertain [3,4].

Current evidence suggests that raTKA improves the accuracy and reproducibility of component positioning and may be associated with improved early functional outcomes compared with mTKA [2,5]. However, clinical findings remain heterogeneous, and long-term comparative data are still limited. Moreover, robotic platforms differ in their planning methods, imaging requirements, registration processes, and intraoperative feedback; therefore, the functional impact of each system should be evaluated independently [6].

Gait analysis provides an objective assessment of postoperative functional recovery and may detect biomechanical differences that are not fully captured by conventional clinical scores or patient-reported outcome measures (PROMs). Nevertheless, research evaluating gait and postural outcomes after raTKA remains limited. Previous studies have investigated functional gait assessment and three-dimensional gait changes after raTKA using other robotic platforms [7,8,9]. To our knowledge, no peer-reviewed study has specifically assessed postoperative gait and postural outcomes after ROSA-assisted TKA.

Therefore, this comparative cohort study aimed to evaluate early gait, postural, functional, and PROMs in patients undergoing ROSA raTKA compared with mTKA. To reduce procedural heterogeneity, all procedures were performed by a single senior orthopaedic surgeon using the same implant and alignment technique. PROMs were additionally compared at the final follow-up.

## 2. Materials and Methods

This prospective, non-randomized comparative cohort study was approved by the Academic Health Research Ethics Board (6.237-29/07/2020), and all patients provided written informed consent before inclusion. Adult patients undergoing primary unilateral TKA for symptomatic end-stage knee osteoarthritis were eligible. Exclusion criteria included revision TKA, complex primary TKA, use of a different implant design, and inability to complete the scheduled functional assessment. The raTKA group comprised consecutive primary unilateral ROSA-assisted TKAs (ROSA Knee System; Zimmer Biomet, Warsaw, IN, USA) performed by a senior orthopaedic surgeon from October to December 2022, using a posterior-stabilised implant (NexGen Legacy LPS Flex, Zimmer Biomet, Warsaw, IN, USA). The mTKA cohort consisted of consecutive primary unilateral manual TKAs performed by the same surgeon during the same study period, using the same implant and perioperative protocol. The same cemented posteriorly stabilised prosthesis (NexGen Legacy LPS Flex, Zimmer Biomet, Warsaw, IN, USA) was consistently used in all cases. Treatment allocation was non-randomised and based on patient preference after discussion of both surgical options. The senior surgeon performs approximately 300 TKAs per year. Robotic procedures were performed after the surgeon completed the initial learning curve.

All patients received uniform perioperative and postoperative care, including general anaesthesia, standard chemoprophylaxis, pain management, and physiotherapy. Anti-thrombotic stockings were used. Mobilisation was commenced within 12 h post-surgery, with full weight-bearing permitted as tolerated. Physiotherapy emphasised early range of motion (ROM) recovery, quadricep activation, progressive strengthening, gait and transfer training, and functional mobility. The identical institutional rehabilitation protocol was implemented for both groups, with no modifications specific to either group.

**Figure 1:**
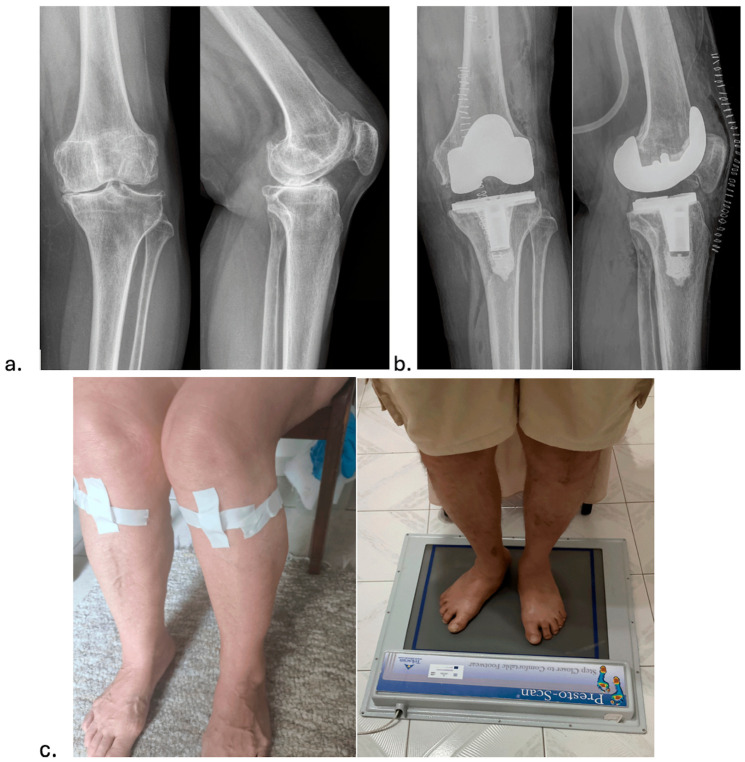
An exemplary patient case showing (**a**) preoperative and (**b**) postoperative knee X-rays of the procedure, which was performed using a gap-balancing technique within the framework of functional alignment surgery, as well as (**c**) pictures from the gait analysis (preoperative sensor placement around the knees and body weight distribution between legs measured with the patient standing barefoot on a posturographic platform.

All procedures were performed using a gap-balancing technique within the framework of functional alignment. The surgical technique was previously published in detail [5]. Resection planning followed a tibia-first approach, starting with the extension gap. The proximal tibial resection was designed to replicate the native medial proximal tibial angle, with varus constraints ranging from 0° to 5°. Slope was matched to the native medial plateau whenever possible. The distal femoral resection was then planned to ensure it was parallel to the tibial resection, resulting in a rectangular extension gap. Overall coronal alignment limits were set between 3° varus and 5° valgus. Additional soft-tissue releases and/or recuts of the distal femur or proximal tibia were performed when necessary to achieve balanced gaps. The rotation of the femoral component was planned to achieve a balanced flexion gap, with lateral laxity 0.5–1 mm greater than medially (i.e., a rectangular-to-slightly trapezoidal gap). Anteroposterior positioning and sizing of the femoral component were adjusted to achieve 1–2 mm mediolateral component laxity. The imageless mode of the ROSA robot was used for robotic cases (Figure 1). In the mTKA group, the same alignment philosophy was applied manually using conventional instrumentation. Thigh tourniquet pressure was applied before the skin incision and maintained until the skin closed.

### 2.1. Outcomes

An experienced physiotherapist, not involved in the surgical procedures, conducted follow-up clinical assessments and analysed gait and posture. The physiotherapist was not involved in the surgical procedures but was not blinded to treatment allocation. Gait, postural, strength, ROM, and functional assessments were performed preoperatively and at three months postoperatively. PROMs, including Knee Injury and Osteoarthritis Outcome Score (KOOS) Pain, activities of daily living (ADL), and quality of life (QoL), were additionally assessed at final follow-up. The primary outcome was postoperative CoM kinematics during walking at three months. Secondary outcomes included quadricep and tibialis anterior MVIS, weight distribution, TUG, ROM, KOOS domains, and complications.

### 2.2. Clinical Outcomes

Before surgery, a comprehensive medical history and demographic information were collected. The physiotherapist assessed knee ROM and patients’ quality of life using the KOOS (Pain, ADL, and QoL scores). Additionally, radiographic follow-up, perioperative medical events, and postoperative complications were recorded. 

### 2.3. Gait and Posture Outcomes

#### 2.3.1. Muscle Strength Evaluation

##### Grip Strength

A grip strength dynamometer (Kinvent, Montpellier, France) was used to test patients’ overall muscle strength. Participants sat with their shoulders at 0° of flexion, elbows at 90°, and forearms in a neutral position. Both hands were tested by squeezing the dynamometer as hard as possible for three repetitions, each held for five seconds with a 10 s rest between squeezes. Finally, the highest value (kg) from the three attempts for each hand was recorded, and the bilateral scores were averaged (kg).

##### Quadricep and Tibialis Anterior Maximum Voluntary Isometric Strength (MVIS)

A hand-held dynamometer (HHD; Muscle Controller, Kinvent, Montpellier, France) was used to assess MVIS. Quadricep strength was measured while seated at a 90-degree knee flexion, and tibialis anterior strength was measured in the supine position with the ankle neutral. Both legs followed the same procedure: the dynamometer was pressed to maximum effort for three repetitions of five seconds each, with a 10 s rest between efforts. The highest value was recorded for each muscle bilaterally across the three attempts.

#### 2.3.2. Body Centre-of-Mass (COM) Kinematics During Walking

The walking pattern was assessed using inertial measurement unit (IMU) sensors (Movesense^TM^, Vantaa, Finland). These sensors utilise a nine-axis IMU to capture movement data, comprising an accelerometer, a gyroscope, and a magnetometer, each with three axes. We linked the IMU sensor to a receiving device during data collection. Each participant was outfitted with a sensor on the sacrum. After setting up the sensor, participants were instructed to walk comfortably at a distance of 4–5 m and return twice. The primary CoM parameter was CoM velocity, expressed in mm/s. The mean value from the walking trials was used for analysis.

#### 2.3.3. Body Weight Distribution Between Legs

Patients stood barefoot on a posturographic platform to assess weight distribution and stability, maintaining a quiet bipedal stance for 10 s. Measuring 700 × 500 mm, the platform featured 2304 resistive sensors with 0.001 kPa accuracy, sampled at 60 Hz. The patients stood in socks, with their feet in a standardised position, aligning their heels with the tape on the platform. The distance between the first metatarsal heads was measured for accurate follow-up. The platform recorded foot pressure distributions using EPS; Foot Checker 4.1 (LorAn Engineering Srl, Castel Maggiore, Bologna, Italy). Weight distribution between the legs was calculated as a percentage of the patient’s overall weight and measured twice, with the eyes either closed or open. 

#### 2.3.4. Time Up-and-Go Test (TUG)

The TUG test is a validated assessment designed to evaluate an individual’s functional mobility and dynamic balance, offering vital insights into their capabilities for daily living [10]. This assessment measures the duration required for a person to rise from a seated position, ambulate three meters at their normal walking pace while using their customary assistive device, pivot, and return to the chair to resume a seated posture [11]. This test involved patients wearing their usual footwear and using a walking aid if necessary. The assessment starts with patients sitting down and standing up upon the therapist’s cue. Each patient walked three meters, turned around a placed cone, returned to the chair, and sat down again. The timing stopped once the patient was seated. A practice trial was conducted before the timed trial [11]. We measured each patient twice: once when they turned the cone left at 3 m and again when they turned the cone right. The average time from the two trials was used for analysis.

### 2.4. Statistical Analysis

The necessary sample size was determined based on reported differences and standard deviations of measurements between mTKA and raTKAs in previous gait analysis studies [7]. Using Lehr’s formula, our statistical analysis indicated that, with a power of 0.8 and an α level of 0.05, at least 24 patients per group needed to be enrolled to detect a significant difference in COM kinematics of 0.1 mm/s between the groups.

Continuous variables were assessed for normality using the Shapiro–Wilk test. Normally distributed data were reported as mean ± standard deviation and compared using independent-samples *t*-tests, whereas non-normally distributed data were reported as median and interquartile range and compared using the Mann–Whitney U test. Categorical variables were reported as frequencies and percentages and compared using the chi-square test. Within-group changes from baseline to three months were assessed using paired *t*-tests or Wilcoxon signed-rank tests.

Multivariable linear regression was performed to assess between-group differences after adjustment for potential confounders. The postoperative value was entered as the dependent variable, treatment group as the independent variable, and baseline value, age, sex, and BMI as covariates. Regression coefficients with 95% confidence intervals were reported. Because multiple secondary outcomes were evaluated, an exploratory Holm–Bonferroni correction was applied to secondary between-group comparisons. Statistical significance was set at *p* < 0.05. Analyses were performed using IBM SPSS Statistics, Version 28.0 (IBM Corp., Armonk, NY, USA).

## 3. Results

A total of 70 patients were included in the analysis, comprising 46 patients in the raTKA group and 24 patients in the mTKA group. No patients were lost to follow-up. The overall cohort included 51 females and 19 males, with a mean age of 73.2 ± 7.1 years and a mean BMI of 31.5 ± 6.1 kg/m^2^. All patients completed the three-month postoperative assessment and the PROMs assessment at the final follow-up. The mean final follow-up was 3.19 ± 0.27 years, with no significant difference between groups (Mann–Whitney test, *p* = 0.689). The two groups were also comparable in terms of age, BMI, sex distribution, grip strength, and preoperative KOOSs (Table 1 and Table 2).

### 3.1. Clinical Outcomes

No intraoperative complications occurred in either group. At three months, mean ROM was comparable between the raTKA (126.5° ± 16°) and mTKA (125.5° ± 14°) groups; Mann–Whitney test, *p* = 0.165. Both groups demonstrated significant improvement in KOOSs by three months, with no significant between-group differences at this time point (Table 2). At final follow-up, after a mean follow-up of 3.2 years, KOOS ADL (*p* = 0.041) and QoL (*p* = 0.032) were significantly higher in the raTKA group than in the mTKA group (Table 2).

### 3.2. Grip Strength

Preoperative grip strength was comparable between the raTKA and mTKA groups: 14.3 ± 11.7 kg versus 14.3 ± 7.2 kg, respectively; Mann–Whitney test, *p* = 0.347. At three months postoperatively, grip strength remained similar between groups: 14.3 ± 6.6 kg versus 14.1 ± 5.2 kg, respectively; Mann–Whitney test, *p* = 0.415.

### 3.3. Quadricep and Tibialis Anterior MVIS

Mean quadricep MVIS improved significantly in the overall cohort by three months postoperatively (Wilcoxon signed-rank test, *p* = 0.042). Preoperatively, quadricep MVIS did not differ significantly between the raTKA group and the mTKA group: 20.0 ± 7.9 versus 20.1 ± 8.5, respectively; Mann–Whitney test, *p* = 0.819. At three months, there was no significant difference in quadricep MVIS between the raTKA (18.6 ± 9) and mTKA (17.9 ± 6.5) groups; Mann–Whitney test, *p* = 0.257.

Tibialis anterior MVIS improved significantly in the overall cohort at 3 months postoperatively (paired-samples *t*-test, *p* < 0.001). Preoperatively, tibialis anterior MVIS was comparable between the raTKA and mTKA groups: 11.6 ± 3.2 versus 12.3 ± 3.4, respectively; Student *t*-test, *p* = 0.504. At three months, tibialis anterior MVIS was comparable between the raTKA and mTKA groups: 13.9 ± 3 versus 12.7 ± 2, respectively; Student *t*-test, *p* = 0.327.

### 3.4. Body COM Kinematics

CoM velocity did not change significantly in the overall cohort by three months postoperatively (Paired sample *t*-test, *p* = 0.423). Preoperatively, mean CoM values were comparable between the raTKA and mTKA groups: 0.13 ± 0.08 mm/s versus 0.14 ± 0.08 mm/s, respectively; Student *t*-test, *p* = 0.905. At three months, CoM values were similar between groups: 0.12 ± 0.07 mm/s versus 0.12 ± 0.05 mm/s, respectively; Student *t*-test, *p* = 0.066. 

### 3.5. Body Weight Distribution

Preoperatively, the mean side-to-side absolute difference in body weight distribution was similar between the raTKA and mTKA groups: 4 ± 18.5% versus 6 ± 8.3%, respectively; Mann–Whitney test, *p* = 0.71. At three months, the raTKA group showed a mean absolute side-to-side difference comparable to that of the mTKA group: 12 ± 19.2% versus 12 ± 6.75%, respectively; Mann–Whitney test, *p* = 0.733.

### 3.6. TUG Test

The mean TUG time decreased significantly in the overall cohort by three months postoperatively (Wilcoxon signed-rank test, *p* < 0.001). Preoperatively, TUG time was comparable between the raTKA and mTKA groups: 11.2 ± 5.9 s versus 11.3 ± 3.6 s, respectively; Mann–Whitney test, *p* = 0.767. At three months, TUG time was lower in both groups, but the between-group difference was not statistically significant: 9.4 ± 3.7 s versus 9.7 ± 3.0 s, respectively (Mann–Whitney test, *p* = 0.599).

### 3.7. Multivariate Regression Analysis and Multiple Secondary Comparisons

In exploratory multivariable linear regression adjusted for age, sex, and BMI, the treatment group was not significantly associated with the primary CoM velocity outcome at three months (β = −0.002 mm/s, 95% CI −0.038 to 0.033; *p* = 0.894). Similarly, no significant adjusted association was observed between treatment group and early objective functional outcomes, including TUG (β = −0.335 s, 95% CI −1.295 to 0.625; *p* = 0.488), quadricep MVIS (β = 1.750, 95% CI −0.176 to 3.676; *p* = 0.074), tibialis anterior MVIS (β = 0.895, 95% CI −0.172 to 1.962; *p* = 0.099), absolute weight-distribution asymmetry (β = 2.504%, 95% CI −3.967 to 8.975; *p* = 0.442), or ROM (β = 2.963°, 95% CI −1.766 to 7.692; *p* = 0.215). At final follow-up, raTKA remained independently associated with higher KOOS ADL (β = 3.192, 95% CI 1.106 to 5.278; *p* = 0.003) and KOOS QoL (β = 3.132, 95% CI 0.253 to 6.011; *p* = 0.033) after adjustment for baseline score, age, sex, and BMI. However, multiple-comparison correction was applied to the secondary between-group comparisons. After Holm–Bonferroni correction, none of the secondary outcomes remained statistically significant (KOOS QoL, final follow-up, *p* = 0.384, KOOS ADL, final follow-up, *p* = 0.451).

## 4. Discussion

To our knowledge, this is the first study to compare early gait, postural, strength, functional, and PROMs between ROSA-assisted TKA and manual TKA. The main finding was that ROSA raTKA and mTKA demonstrated comparable early objective functional recovery at three months. No measurable between-group difference was detected in early objective gait, postural, strength, ROM, or TUG outcomes. The marginal unadjusted differences observed in the selected final follow-up KOOS domains should be interpreted as exploratory only, as they did not remain significant after correction for multiple secondary comparisons. The short follow-up and small sample size may have limited the ability to detect differences, but the present data do not demonstrate objective superiority of ROSA-assisted TKA. Therefore, the hypothesis that ROSA raTKA improves early gait and postural outcomes compared with mTKA remains plausible but unproven.

Previous studies have shown that raTKA can improve the accuracy and reproducibility of implant positioning compared with conventional instrumentation. However, whether improved technical accuracy translates into superior functional recovery remains uncertain [1,2,5]. Existing evidence on gait after raTKA remains limited [7,8,9]. A recent randomized trial comparing raTKA with mTKA found no major between-group differences in cadence, walking velocity, or plantar pressure ratios at 12 months, although selected parameters, including propulsion time and lateral sway, favored raTKA [7]. These findings are consistent with the present study, in which early objective gait and postural parameters were broadly similar between groups.

The ROSA Knee System has been evaluated in studies that primarily focus on implant positioning accuracy, early clinical outcomes, and PROMs [2]. Hasegawa et al. compared NAVIO and ROSA raTKA and reported comparable implantation accuracy between systems, with limited differences in early clinical scores [12]. A recent propensity-matched study of ROSA functionally aligned rTKA found equivalent satisfaction and PROMs compared with manual mechanically aligned TKA at six months [13]. In this context, the present study adds objective gait, postural, and strength measurements to the available ROSA literature.

Muscle strength recovery is an important determinant of postoperative function after TKA. Advanced knee osteoarthritis causes stiffness, muscle atrophy and weakness, and impaired quadricep activation [14,15,16]. Wang et al. found that muscle strength and mobility improved by the third month post-TKA, but these improvements remained below those of non-arthritic individuals [14]. In the present study, quadricep and tibialis anterior MVIS improved significantly in the overall cohort by three months. No measurable between-group difference was detected in the quadricep and tibialis anterior MVIS. These findings suggest that both surgical approaches were associated with early strength recovery, while any potential advantage of raTKA in muscle recovery requires confirmation in larger cohorts.

Body weight distribution and postural control are clinically relevant after TKA because impaired balance may persist despite improvement in pain and conventional clinical scores. Postural stability issues contribute to falls in older adults [17,18]. In osteoarthritis, impaired stability results from proprioceptive deficits, muscle weakness, and knee pain [19]. Knee osteoarthritis patients exhibit increased postural sway, greater anterior–posterior sway during sit-to-stand transitions, and reduced control when shifting from a double-leg to a single-leg position [20]. While TKA improves functional scores, balance issues linked to falls may persist [17]. In this study, the raTKA group showed a comparable absolute side-to-side difference in body weight distribution to the mTKA group at three months. The data do not demonstrate superior postural recovery with raTKA, and further evaluation may be useful in future raTKA studies.

CoM kinematics provide an objective assessment of whole-body movement during ambulation and may detect functional compensation not captured by standard PROMs. Measuring COM trajectories in natural settings shows how gait and balance change with disease [21], improving understanding of their impact on daily life and management. Naili et al. demonstrated the COM trajectory as a sensitive indicator of functional compensation after TKA in patients with knee osteoarthritis during a sit-to-stand test [22]. In the present study, CoM velocity at three months was comparable between raTKA and mTKA groups. This finding suggests comparable early gait recovery between ROSA raTKA and mTKA. Longer follow-up may be required to determine whether subtle differences in gait adaptation emerge after the early recovery phase.

The TUG test improved significantly in the overall cohort by three months, reflecting improved functional mobility after TKA. Other TKA studies report similar improvements in the TUG test [10]. Healthy individuals aged 60–80 usually complete the TUG in 10 s, with older adults taking 1–2 s longer [23]. The TUG test reliably measures function, balance, and walking in patients with knee osteoarthritis, making it a widely used, dependable performance measure for TKA [11,24,25]. A 2.27 s change in TUG signals a significant shift in post-TKA rehab, suggesting those without this progress may need more rehabilitation [11]. In our study, the average TUG improvement was approximately 2.2 s and was not different between the two groups. This supports the interpretation that both techniques produced meaningful early functional improvement, without clear evidence of superiority for either approach in early dynamic balance.

The significantly better KOOS ADL and QoL at final follow-up observed in the raTKA group should be interpreted with caution, as they did not remain significant after correction for multiple secondary comparisons. Patient mid-term PROM differences after TKA are multifactorial and may be influenced by pain relief, expectations, confidence in the surgical technology, perceived recovery, and unmeasured functional parameters. Because most objective gait and postural outcomes did not differ significantly between groups, the present study cannot conclude that better PROMs were directly attributable to superior gait or posture. Further studies with larger randomised cohorts and longitudinal gait assessments are needed to clarify the relationship among robotic assistance, objective functional recovery, and patient-perceived outcomes.

Several limitations should be considered. First, the study had short-term follow-up for objective gait and postural outcomes, and longer follow-up is required to determine whether early numerical differences persist or become clinically relevant. Second, the study was non-randomized, with treatment allocation based on patient preference, introducing potential selection and expectation bias. This is particularly relevant because the between-group differences observed in selected PROM domains were subjective outcomes, whereas the objective gait, postural, strength, ROM, and TUG measures were comparable between groups. Patients choosing raTKA may have different expectations, perceptions of advanced technology, confidence in treatment, motivation for rehabilitation, or socioeconomic characteristics, all of which could influence satisfaction and PROMs independently of objective functional recovery. Third, the physiotherapist was not blinded to treatment allocation. Although several instrumented measurements were included, lack of assessor blinding may have influenced subjective and performance-based outcomes, including satisfaction assessment and the TUG test. Therefore, small between-group differences in these outcomes should be interpreted cautiously. Fourth, although an a priori sample-size estimate was performed, the final cohort was small, particularly in the mTKA group, limiting statistical power for secondary outcomes. Fifth, only selected gait and postural parameters were assessed; more comprehensive three-dimensional gait analysis could provide additional information on knee kinematics, kinetics, and compensatory movement patterns. The gait analysis methodology was limited to selected global parameters obtained using a single sacral-mounted IMU. Although this approach allowed objective assessment of CoM velocity, it did not provide a comprehensive biomechanical evaluation of gait. Spatiotemporal and kinematic variables such as gait speed, cadence, step length, stance and swing phase distribution, symmetry indices, knee joint kinematics, and kinetic parameters were not assessed. Therefore, subtle gait adaptations or compensatory movement patterns may have been missed, and future studies should incorporate comprehensive three-dimensional gait analysis. The single-surgeon, single-centre design also limits generalizability. Finally, detailed preoperative and postoperative radiographic alignment parameters, including hip-knee angle, medial proximal tibial angle, lateral distal femoral angle, joint-line restoration, and alignment outlier rates, were not systematically evaluated. Therefore, although both groups were treated according to the same functional-alignment philosophy, the present study cannot determine whether robotic assistance resulted in more accurate restoration of patient-specific alignment or whether alignment differences influenced functional or patient-reported outcomes. Future studies combining comprehensive alignment analysis with objective gait assessment are required to clarify this relationship.

Despite these limitations, the study has several strengths. All procedures were performed by a single experienced surgeon using the same implant, alignment philosophy, perioperative protocol, and rehabilitation pathway, thereby creating a highly standardised setting and reducing procedural heterogeneity. In addition, the study incorporated objective functional assessments, including strength testing, CoM analysis, weight distribution, and the TUG, alongside PROMs.

## 5. Conclusions

In conclusion, ROSA raTKA and mTKA showed comparable early objective gait, postural, strength, and functional recovery at three months. In exploratory analyses, selected final follow-up KOOS domains were marginally higher in the raTKA group, but these differences were no longer significant after multiple-comparisons correction. The objective early gait and postural findings do not demonstrate the superiority of ROSA raTKA. Randomised studies with longer follow-up and more comprehensive gait analysis are needed to determine whether raTKA provides clinically meaningful functional advantages over mTKA.

## Figures and Tables

**Table 1:** A comparative analysis of the baseline characteristics between the mTKA and raTKA groups.

Characteristics		Groups		*p*
		mTKA	raTKA	
Age *		72.6 (7.0)	73.5 (7.2)	0.766 ^&^
Sex ***	**Male**	4	15	0.155 ^#^
	**Female**	20	31	
BMI **		31.3 (6.2)	31.9 (7.2)	0.205 ^@^
Normal weight ****		1 (4.2)	0 (0)	0.372 ^#^
Overweight ****		7 (29.2)	13 (28.3)	
Obese ****		16 (66.7)	33 (71.7)	

* The values are presented as the mean with the standard deviation in parentheses; ** The values are presented as the median with the interquartile range in parentheses; *** The values are provided as raw numbers; **** The values are provided as raw numbers with the percentages in parentheses; ^&^ Tests were conducted using Student’s *t*-test; ^@^ Tests were conducted using the Mann–Whitney test; ^#^ Tests were conducted using the Chi-squared test; mTKA: manual total knee arthroplasty, raTKA: robotic total knee arthroplasty, BMI: body mass index.

**Table 2:** A comparative analysis of the mean scores pertaining to the KOOS domains between mTKA and raTKA groups.

Characteristics	Groups		*p*
	mTKA	raTKA	
*preoperative KOOS*			
Pain *	56.9 (16.8)	57.0 (19.6)	0.252 ^&^
ADL *	50.9 (19.2)	47.7 (16.7)	0.250 ^&^
QoL **	25 (17.1)	28.1 (25)	0.540 ^@^
*postoperative KOOS (3 months)*			
Pain **	90.2 (18.0)	91.6 (16.6)	0.681 ^@^
ADL **	86.7 (20.5)	88.2 (16.5)	0.581 ^@^
QoL **	62.5 (42.1)	75 (37.5)	0.506 ^@^
*postoperative KOOS (last follow-up)*			
Pain **	91.6 (7.6)	97.2 (8.3)	0.271 ^@^
ADL **	91.1 (7.3)	94.1 (7.8)	0.041 ^@^
**QoL**	87.5 (6.2)	93.7 (6.2)	0.032 ^@^

* The values are presented as the mean with the standard deviation in parentheses; ** The values are presented as the median with the interquartile range in parentheses; ^@^ Tests were conducted using the Mann–Whitney test; ^&^ Tests were conducted using Student’s *t*-test; mTKA: manual total knee arthroplasty, raTKA: robotic total knee arthroplasty, BMI: body mass index, ADL: activities of daily life, QoL: quality of life.

## Data Availability

The raw data supporting the conclusions of this article will be made available by the authors on request.

## Abbreviations

The following abbreviations are used in this manuscript:

raTKA	robotically assisted total knee arthroplasty
ROSA	Robotic Surgical Assistant
PROMs	patient-reported outcome measures
mTKA	manual total knee arthroplasty
MVIS	maximum voluntary isometric strength
CoM	centre of mass
TUG	Timed Up-and-Go
ROM	range of motion
KOOS	Knee injury and Osteoarthritis Outcome Score
ADL	activities of daily living
QoL	quality of life
BMI	body mass index
IMU	inertial measurement unit
HHD	hand-held dynamometer
SPSS	Statistical Package for the Social Sciences
